# A New Model for Teaching Radiological Anatomy

**DOI:** 10.1002/ca.70092

**Published:** 2026-02-10

**Authors:** James Coey, Thomas Connolly, Ingrid Gouldsborough, Matthew Jones, Bipasha Choudhury

**Affiliations:** ^1^ Faculty of Biology, Medicine and Health The University of Manchester Manchester UK

**Keywords:** clinical teaching fellows, computed tomography, medical education, medical licensing assessment, radiological anatomy, undergraduate curriculum, X‐ray interpretation

## Abstract

The General Medical Council (GMC) and the Royal College of Radiologists (RCR) Undergraduate Radiology Curriculum emphasize the need for medical graduates to use anatomical knowledge when interpreting imaging studies. This study evaluated a model in which Clinical Teaching Fellows (CTFs) were upskilled to deliver radiologist‐designed tutorials using computed tomography (CT) imaging to facilitate the identification of key anatomical landmarks on chest and abdominal X‐rays. Two tutorials, aligned with our institution's pre‐clinical curriculum, were developed by radiology residents and anatomy faculty for 430 first‐year and 420 s‐year medical students. CTFs were trained using structured pre‐learning resources and then facilitated small‐group sessions where students interacted with CT scans and correlated the anatomy with X‐rays. Feedback was collected from students and tutors. Response rates were high (76% first year; 88% second year). Most students (87%) reported feeling prepared, 94% found sessions enjoyable, and nearly all (99.9%) found CT imaging useful for learning X‐ray anatomy. Among tutors (*n* = 11), confidence in teaching with CT imaging rose significantly, with those reporting themselves as quite or very confident increasing from 28% to 91%. Tutors also reported improved confidence in viewing CT scans in their own clinical practice. Radiological anatomy teaching can therefore be delivered sustainably through the upskilling of non‐radiologist educators. This model enhances student understanding of clinically relevant anatomy, aligns with national guidance, and provides professional development benefits for tutors. It offers a pragmatic strategy to integrate radiology into undergraduate curricula at scale.

## 1 Introduction

In 2024, the General Medical Council (GMC) introduced the “Medical Licensing Assessment” (MLA), a national examination undertaken by all final‐year medical students. The MLA is designed to ensure that every new doctor meets a consistent standard of safe clinical practice (General Medical Council 2021). To support this, the MLA Content Map was published, outlining the core knowledge, skills and behaviors expected of graduating students (General Medical Council 2021). Notably, clinical imaging is identified as a key “Area of Clinical Practice,” with clear expectations for students to recognize common conditions on imaging (General Medical Council 2021).

In response, the Royal College of Radiologists (RCR) released the “Undergraduate Radiology Curriculum,” offering a structured approach to integrating Clinical Radiology into UK medical education (Royal College of Radiologists 2022). In particular, the curriculum draws attention to the link between anatomical knowledge and the interpretation of imaging studies, including X‐rays and computed tomography (CT) scans (Royal College of Radiologists 2022).

The mutually beneficial relationship between anatomy and radiology is already well established; as early as 1927, educators noted the value of integrating imaging alongside cadaveric dissection, observing that it helps students “think of the structures found in the cadaver as in place in the living body” (Bardeen 1927). Studies consistently show that early exposure to radiological anatomy improves examination performance and builds student confidence in image interpretation (Chew et al. 2020; Ekelund and Elzubeir 2000; Bell et al. 2019). Furthermore, the interpretation of medical images and the use of image‐guided procedures are no longer exclusive to radiologists. Today, physicians across a wide range of specialties are expected to incorporate imaging into their routine practice, and this requirement is reflected in the MLA content map. For example, emergency physicians may use point‐of‐care ultrasound, anesthetists often place intravascular lines under imaging guidance, and neurosurgeons routinely interpret CT and magnetic resonance imaging (MRI) scans as part of their clinical decision‐making.

Recognizing the growing importance of radiological anatomy in the context of modern clinical practice, the RCR now recommends that all UK medical schools incorporate radiologist‐led imaging teaching into their existing anatomy curricula. This integration offers clear educational benefits with a focus on clinically‐relevant anatomy, yet significant barriers remain—including the constraints of already pressured undergraduate timetables and a projected 40% shortfall in consultant radiologists over the next five years (Royal College of Radiologists 2022, 2024).

At our institution, our pre‐clinical medical curriculum (Years 1 and 2) follows a Team‐Based Learning (TBL) model. Each week is structured around a clinical theme that informs lectures, laboratory sessions, and discussions, culminating in a synthesis of key learning points at the end of the week. Anatomy is fully integrated into this framework, with a typical week consisting of a 1‐h small group classroom‐based tutorial followed by a 1‐h dissecting room session involving interaction with prosections and anatomical models, living anatomy and cadaveric dissection. These sessions are supplemented by whole‐cohort lectures where relevant. A team of 20 full‐time Clinical Teaching Fellows (CTFs)—typically resident doctors who have completed foundation training and are preparing for specialist training—supports the delivery of the anatomy content under the supervision of the senior anatomy faculty. CTFs take up such educational posts for 9 months before returning to clinical practise.

Although students already receive some exposure to X‐ray imaging as part of the existing anatomy curriculum, this study aimed to enhance and formalize the teaching of radiological anatomy in line with the RCR guidance. Small‐group tutorials were identified as the most effective way of delivering this content, however, given the large cohort of students at our medical school, delivering this content directly via radiologists across multiple parallel teaching sessions was not feasible. Instead, we adopted a novel and scalable approach by upskilling a cohort of CTFs to deliver radiologist‐designed tutorials to our large student body. Specifically, the use of CT imaging was used to facilitate the identification of key anatomical landmarks on common X‐ray images, ensuring alignment with the RCR undergraduate curriculum requirement for all medical students to apply anatomical knowledge in the interpretation of chest and abdominal X‐rays.

## 2 Materials and Methods

### 2.1 Session Design

Two small‐group tutorial sessions were developed collaboratively by two specialist trainee (ST3) Radiology Residents and the anatomy faculty at our institution. The first session focused on thoracic anatomy and was designed for a cohort of 430 first‐year medical students, while the second, focusing on abdominal anatomy, was designed for 420 s‐year students. The aim of the tutorials was to introduce CT imaging as a conceptual bridge between students' foundational anatomical knowledge and their interpretation of chest and abdominal X‐ray anatomy, respectively.

Sessions were delivered by our existing CTFs in a classroom‐based format, with groups of 10–12 pre‐clinical students. The content of the thoracic and abdominal CT tutorials was aligned with the weekly themes of the TBL curriculum, and the sessions replaced the usual weekly small‐group anatomy tutorials. Although the tutorials themselves did not incorporate specimens, they were intentionally scheduled at the end of six weeks of thoracic and abdominal anatomy teaching, respectively, during which students learned the relevant material in small‐group tutorials and examined and handled the corresponding specimens in the dissecting room.

During the sessions, students worked in small groups using their own electronic devices to interact with normal CT scans via a cloud‐based Picture Archive and Communication Server (PACS). The students were provided with a worksheet containing a series of activities focused on identifying clinically relevant anatomy in multiple imaging planes, followed by correlation with corresponding X‐rays. For example, students were asked to trace the trachea in axial and coronal views on a thoracic CT scan and then locate its course and bifurcation on a frontal chest X‐ray.

To support these sessions, an “Introduction to CT” video was created as required pre‐learning for students to view in advance. This 10‐min video explained how CT scans are acquired using X‐ray radiation, introduced the concepts of CT density and windowing, and re‐visited the different anatomical planes in an imaging context.

### 2.2 Tutor Training

A lesson plan was created for the tutors to use during the session. This included an overview of the session, a list of the required equipment and estimated timings for each section. Student learning objectives were developed in conjunction with the anatomy faculty and included on the lesson plan for the tutors.

To support the delivery of the session, an asynchronous 6‐min tutorial video was created for the CTFs to watch. This was a screen recorded walkthrough of the PACS viewer, demonstrating the features of the software which they would need to operate during the session, and the key anatomical structures on the CT scan that the students would be asked to find.

The CTFs were then given access to the cases in advance to familiarize themselves with the software and anatomical structures. They also had access to “IMAIOS” (an online radiological atlas) and were invited to contact the Radiology Residents should they have any queries prior to the session.

### 2.3 Post‐Session Feedback

Post‐session feedback was collected from both the students and tutors. The Qualtrics survey platform was used to collect and collate the feedback. The questionnaires were designed in conjunction with the senior anatomy faculty, who reviewed each question for its validity. No formal validity or reliability assessments were conducted.

The students' questionnaire contained 13 questions, and this feedback was collected prior to the end of each session. Using a mix of Likert scale questions and free text responses, the survey aimed to evaluate the students' prior exposure to radiology, the effectiveness of the pre‐recorded lecture and the usefulness of the session. Student demographic data was not collected as part of the survey.

The tutor feedback questionnaire contained 12 questions using a mix of Likert scale questions and free‐text responses, aiming to assess the tutors' previous experience with teaching using radiology, the effectiveness of the pre‐session materials, and their evaluation of the session. Given that multiple sessions were conducted over an entire week, tutor feedback was collected retrospectively.

Feedback was analyzed using Microsoft Excel.

Ethical approval was sought but the study was deemed exempt by our institution's ethics committee as it met the criteria for an educational audit.

## 3 Results

### 3.1 Student Evaluation

#### 3.1.1 Response Rate

Responses were received from 329 of 430 first‐year medical students (76%) for the thoracic CT session evaluation, and from 370 of 420 s‐year medical students (88%) for the abdominal CT session evaluation.

#### 3.1.2 Previous Radiology Exposure

**FIGURE 1:**
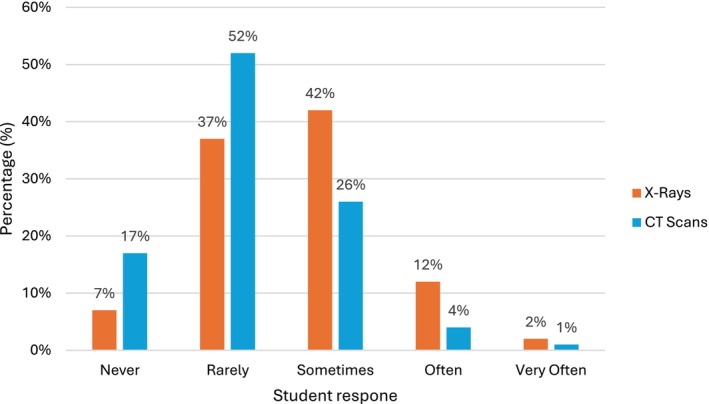
How frequently do you look at the following imaging modalities in your studies?

Students perceived their exposure to radiology to be minimal. In total across both sessions, 97 of 669 students (15%) said they *often* or *very often* look at X‐ray images in their studies, with 310 of 699 students (44%) stating they *rarely* or *never* do. Only 39 of 699 students (6%) said they *often* or *very often* look at CT scans, with 478 of 699 students (68%) *never* or *rarely* looking at them (Figure 1).

#### 3.1.3 Preparedness for the Session

**FIGURE 2:**
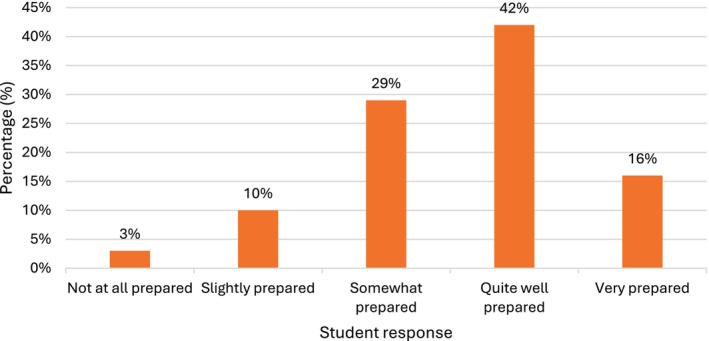
How well prepared for the session did you feel after watching the “Introduction to CT” video?

Most students (609/699, 87%) reported feeling at least *somewhat* prepared for the teaching session after watching the “Introduction to CT” video, with over half (404/699, 58%) feeling *quite well* or *very well prepared*, indicating that the video was generally useful in supporting session readiness (Figure 2).

#### 3.1.4 Session Evaluation

The session was rated as *quite* or *very enjoyable* by 655 of 699 students (94%), indicating high overall satisfaction. Notably, none of the students reported that the session was *not enjoyable at all*.

**FIGURE 3:**
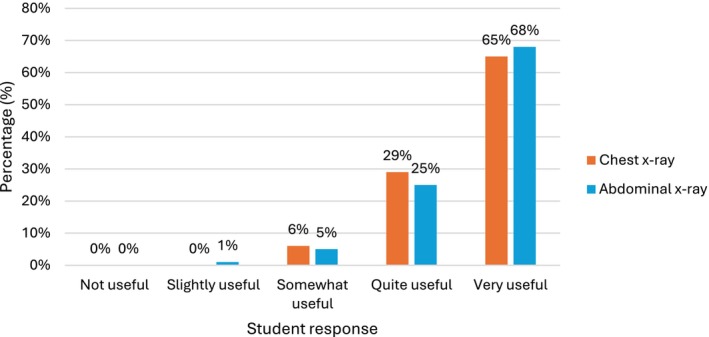
How useful is looking at a CT scan for learning X‐ray anatomy?

All 329 first‐year students reported that reviewing a CT thorax was useful for learning anatomy on chest X‐rays, with the majority (308/329, 94%) rating it as *quite* or *very useful* (Figure 3). Likewise, almost all students (369/370, 99.7%) found reviewing a CT abdomen useful for learning abdominal X‐ray anatomy, and most (345/370, 93.2%) rated it as *quite* or *very useful* (Figure 3).

**FIGURE 4:**
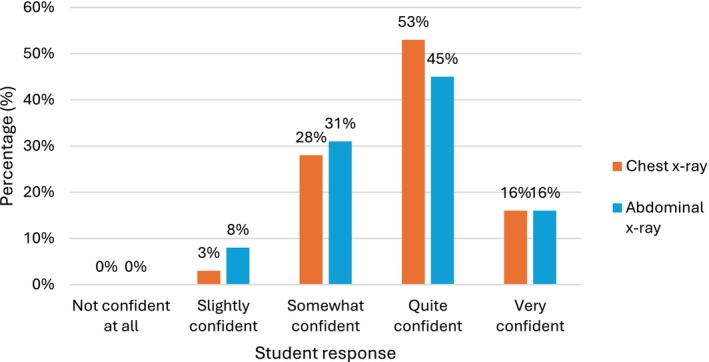
How confident do you now feel to identify anatomical structures on a normal X‐ray?

Following the session, the majority of students reported high levels of confidence in identifying anatomy on X‐rays: 227/329 (69%) felt *quite* or *very confident* with chest X‐rays, and 226/370 (61%) felt *quite* or *very confident* with abdominal X‐rays. Notably, none of the students reported feeling *not confident at all* in identifying anatomy on either modality (Figure 4).

Free‐text student feedback is summarized into six key themes with exemplar quotes in Table 1.

**TABLE 1:** Summary table of free‐text answers to the question “Which elements of the session did you find most useful and why?”—Combined thoracic and abdominal sessions.

Theme	Description	Illustrative quotes
1. Interactive Imaging Practice	Participants highly valued the ability to scroll through CT scans, change views, and explore anatomy dynamically.	“The different options to focus on things in the CT scan. Like looking at the bones and soft tissue separately.” “Looking at the CT scans and being able to scroll through was very useful in improving my anatomical knowledge of the abdomen”
2. Clear Visual & Written Resources	High‐quality CT images, clear markings, and paper handouts helped participants follow along and retain information.	“The interactive resource really helped my orientation and familiarising myself with the software” “The paper handout was really helpful and clear”
3. Real‐life Clinical Relevance	Seeing real patient scans helped bridge theory with practice.	“It was useful for clinical and real‐life application and to help consolidate information. It was nice to be able to visualise the organs in relation to each other in an alive patient.” “Seeing real images of the x‐rays was very useful”
4. Spatial & Orientation Skills	Exercises on orientation and anatomical relationships improved participants overall understanding.	“Looking at the depth of organs in relation to each other” “Figuring out how to orientate myself and using the landmarks to understand the images”
5. Structured Learning Approach	Step‐by‐step guidance, systematic frameworks, and ordered content supported deeper learning.	“I enjoyed the step‐by‐step instructions on how to locate useful organs and also how we got to observe all 3 planes.”
6. Tutor Quality	Participants appreciated knowledgeable tutors, clear explanations, and supportive facilitation.	“My tutor's teaching was unbelievable. I really enjoyed looking at the different structures as it really helped me understand where things are (particularly the coeliac trunk in relation to the lumbar vertebrae)” “Very useful to identify the structures and talk through it with teams and then with the tutor. Really helped to visualise previously abstract concepts.”

### 3.2 Tutor Evaluation

#### 3.2.1 Response Rate and Tutor Experience

A total of 11 tutors led the sessions (6 for the thoracic and 5 for the abdominal CT sessions). All tutors responded to the survey. Of these, 9 of 11 tutors (82%) had been in a teaching role for one year or less. When asked about their use of medical imaging in previous teaching, 4 of 11 tutors (37%) reported sometimes incorporating it, 6 of 11 tutors (55%) reported *rarely* incorporating it, and 1 of 11 (9%) reported *never* incorporating it. Among tutors who had previously used medical imaging in their teaching, 10 of 10 (100%) had used X‐rays, 4 of 10 (40%) had used CT scans, and 2 of 10 (10%) had used ultrasound.

#### 3.2.2 Preparedness for the Session

All 11 tutors found the pre‐session video walkthrough helpful in preparing for the session. Of these, 8 of 11 tutors (73%) felt that the video alone was sufficient in helping them prepare to deliver the session. Just 3 of 11 tutors (27%) stated they would have liked a drop‐in session with a radiologist to answer additional queries.

#### 3.2.3 Session Evaluation

**FIGURE 5:**
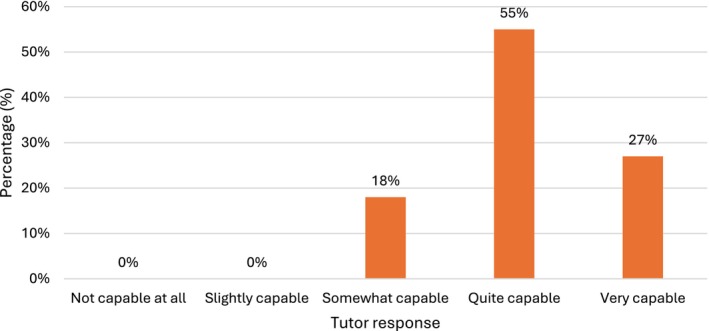
Were students capable of viewing a CT scan and recognizing various anatomical structures?

All 11 tutors felt that CT imaging was helpful in overtly relating the anatomy they taught using dissection, prosections and models to that displayed on X‐ray images and CT scans, with 10 of the 11 (91%) stating that it was either *quite* or *very useful*. Furthermore, tutors felt that students were capable of interpreting CT scans to recognize anatomical structures: 9 of 11 (82%) rated students as either *quite* capable or *very* capable. None of the tutor's felt students were *not at all* or only *slightly capable* (Figure 5).

**FIGURE 6:**
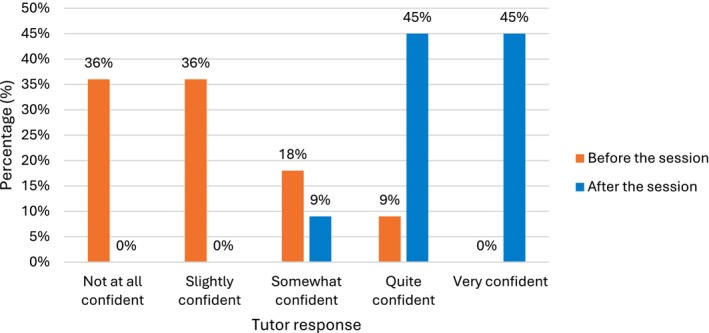
Tutor confidence in teaching anatomy to medical students using CT imaging.

Following the session, there was a substantial increase in tutors' confidence in teaching anatomy using CT imaging. Before the session, 8 of 11 tutors (72%) reported being *not at all* or *slightly confident*, and 0 of 11 reported feeling *very confident*. After the session, 10 of 11 tutors (91%) rated themselves as either *quite confident* or *very confident* (Figure 6).

#### 3.2.4 Wider Clinical Impact

**FIGURE 7:**
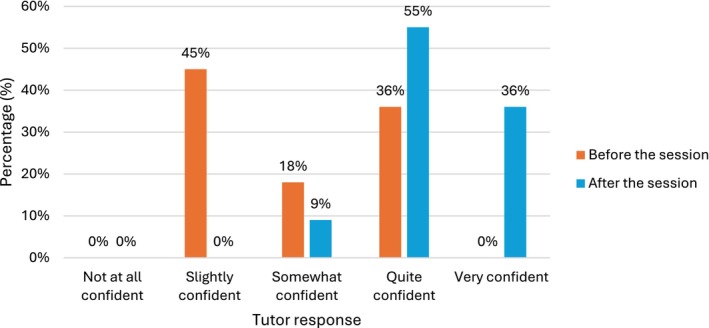
Tutor confidence in opening and viewing a CT scan in the clinical environment.

In addition to increased confidence in teaching using CT scans, tutors also reported an increased confidence in opening and viewing CT scans in the clinical environment in their own practise. Before the session, only 4 of 11 tutors (36%) reported being *quite confident*, and 0 of 11 reported feeling *very confident*. After the session, 10 of 11 tutors (91%) rated themselves as either *quite* or *very confident* (Figure 7).

## 4 Discussion

This study demonstrates that radiologist‐led, imaging‐informed, anatomy teaching can be successfully integrated into the existing anatomy curriculum at our institution, in line with RCR guidance. Importantly, this teaching can be delivered by non‐specialist CTFs following targeted upskilling through a combination of pre‐recorded video tutorials and written materials, thereby reducing the reliance on radiologist direct contact time. Once established, these sessions can be repeated as required and scaled to accommodate large student cohorts (430 students per year in our institution). With appropriate local adaptation to different teaching and learning models, this approach is likely to be transferrable to other medical schools.

Evidence suggests that integrating two‐dimensional with three‐dimensional anatomical representations supports the development of spatial understanding (Anderson et al. 2019). Although students in our sessions did not handle anatomical specimens while reviewing radiologic images contemporaneously, they had encountered the relevant specimens during the immediately preceding teaching period. In addition, because CT is inherently a three‐dimensional modality, students were able to manipulate the CT images across multiple planes, thereby strengthening their grasp of spatial relationships among anatomical structures and their identification of these on two‐dimensional X‐rays. Teaching anatomical relations using different anatomical planes is already a well‐established approach (Weatherall 2006; Standring 2021). A randomized crossover study by Rajprasath et al. (2020) evaluated the effectiveness of integrating radiological and cross‐sectional anatomy into the curriculum for first‐year medical students. The study found that cross‐sectional imaging is useful to highlight and review areas where students' anatomical knowledge is insufficient. Our findings are consistent with this literature: all tutors who delivered the session reported that CT was helpful in relating anatomy taught using dissection, prosections and models to that displayed on X‐ray images. Collectively, these results suggest that CT imaging serves as a valuable educational bridge between existing anatomical teaching methods at our institution and the understanding of radiological anatomy on X‐rays.

While the RCR undergraduate curriculum sets relatively limited expectations for graduating medical students regarding CT image interpretation—specifically, the identification of intracranial hemorrhage and stroke on CT brain scans—it does require that students demonstrate the ability to apply anatomical knowledge effectively when interpreting chest and abdominal X‐rays (Royal College of Radiologists 2022). In particular, students are expected to confidently identify normal anatomical appearances (Royal College of Radiologists 2022). Proficiency in recognizing such normal findings on X‐ray imaging is fundamentally underpinned by a thorough understanding of the relevant anatomy. The radiology tutorials conducted at our institution received high levels of student satisfaction and were associated with increased confidence in identifying anatomical structures on both chest and abdominal X‐rays following review of the corresponding CT images. The absence of demographic data from respondents may hinder the ability to assess for selection bias, however, the overall size of the eligible cohort (*n* = 850) and the high survey response rate (81%) provide some reassurance that the sample is broadly representative of the student population.

An important additional benefit of our approach is the professional development of the CTFs. A review of medical imaging education opportunities for resident doctors and non‐radiologist clinicians highlighted a clear need for enhanced radiology teaching across all levels of training, from undergraduate medical students through to non‐radiologist specialists (Ayesa et al. 2021). Furthermore, the RCR emphasizes that a thorough understanding of normal radiological anatomy is essential for identifying pathological findings (Royal College of Radiologists 2025). Indeed, anatomy forms the foundation of the first postgraduate examination undertaken by Specialist Radiology trainees. By enhancing our tutors' knowledge of cross‐sectional radiological anatomy, they reported increased confidence in opening and viewing CT images in the clinical environment—potentially improving their ability to recognize urgent abnormalities prior to the availability of a formal radiology report.

Feedback from both tutors and students on the asynchronous video tutorials was overwhelmingly positive. While this indirect model of radiologist‐led teaching is essential for delivering content to a large student cohort, it inherently reduces opportunities for face‐to‐face interaction with specialist radiologists. Notably, 3 of the 11 tutors indicated they would have welcomed a drop‐in session with a Radiology Resident to address specific queries. Although all tutors were offered direct email access to the Radiology Residents, no emails were received, suggesting that incorporating a structured in‐person component may better facilitate interaction and address outstanding questions. Despite this, students frequently remarked on the consistently high quality of the tutors' teaching; inter‐tutor variability may have influenced outcomes and introduced some confounding, however all tutors had equivalent post‐qualification experience and delivered the sessions using the same prescriptive teaching materials, which would be expected to mitigate such variability.

These sessions were a planned curriculum intervention for our programme, and therefore lacked a control group. Although piloting the tutorials with a small cohort was theoretically possible, a central aim of the study was to determine whether this instructional approach could be scaled within a large medical school, and under these circumstances, introducing new curricular content to only a proportion of the student body would have been unethical. This may misrepresent the results, especially as the novelty of the sessions may have contributed to the perceived increase in student confidence. It is also worth noting that all student outcomes were self‐reported rather than objectively assessed; consequently, the findings reflect students' *perceived* learning gains and educational value of the intervention, rather than directly measured improvements in performance.

Building on the success of these sessions, further tutorials have been developed to cover neuroanatomy using brain MRI and bony landmarks using X‐rays. In response to student feedback, relevant pathology has been incorporated to enhance anatomical understanding—for example, using hydrocephalus to illustrate the ventricular system, or fractures to highlight bony landmarks. Further objective evaluation is needed to determine the impact of all these sessions on students' performance in summative anatomy assessments and eventually the impact on their clinical practise.

## 5 Conclusion

This study demonstrates that radiological anatomy teaching can be delivered at scale through the upskilling of non‐radiologist CTFs, in accordance with RCR guidance. This novel approach in using CT imaging to facilitate X‐ray interpretation increased student confidence in recognizing anatomical landmarks on chest and abdominal X‐rays. Importantly, this approach not only has the potential to enhance student learning but also contributes to the professional development of CTFs, addressing broader educational gaps identified across the medical training continuum.

This model presents a pragmatic strategy to extend radiologist input across large student cohorts. It aims to improve foundational radiological skills early in medical education, whilst simultaneously augmenting clinical anatomy teaching. Ongoing expansion of this approach to include additional anatomical regions and relevant pathologies offers promising opportunities for further curriculum enhancement. Future studies will be important to assess the impact of this teaching model on summative academic performance and clinical application.

## Ethics Statement

The authors have nothing to report.

## Data Availability

The data that support the findings of this study are available from the corresponding author upon reasonable request.
